# Semi-automated construction of patient individualised clinical target volumes for radiotherapy treatment of glioblastoma utilising diffusion tensor decomposition maps

**DOI:** 10.1259/bjr.20190441

**Published:** 2020-03-19

**Authors:** Roushanak Rahmat, Frederic Brochu, Chao Li, Rohitashwa Sinha, Stephen John Price, Raj Jena

**Affiliations:** 1 Department of Clinical Neuroscience, University of Cambridge, Cambridge, UK; 2 Oncology Centre, Addenbrooke's Hospital, Cambridge, UK

## Abstract

**Objectives:**

Glioblastoma multiforme (GBM) is a highly infiltrative primary brain tumour with an aggressive clinical course. Diffusion tensor imaging (DT-MRI or DTI) is a recently developed technique capable of visualising subclinical tumour spread into adjacent brain tissue. Tensor decomposition through *p* and *q* maps can be used for planning of treatment. Our objective was to develop a tool to automate the segmentation of DTI decomposed *p* and *q* maps in GBM patients in order to inform construction of radiotherapy target volumes.

**Methods:**

Chan-Vese level set model is applied to segment the *p* map using the *q* map as its initial starting point. The reason of choosing this model is because of the robustness of this model on either conventional MRI or only DTI. The method was applied on a data set consisting of 50 patients having their gross tumour volume delineated on their *q* map and Chan-Vese level set model uses these superimposed masks to incorporate the infiltrative edges.

**Results:**

The expansion of tumour boundary from *q* map to *p* map is clearly visible in all cases and the Dice coefficient (DC) showed a mean similarity of 74% across all 50 patients between the manually segmented ground truth *p* map and the level set automatic segmentation.

**Conclusion:**

Automated segmentation of the tumour infiltration boundary using DTI and tensor decomposition is possible using Chan-Vese level set methods to expand *q* map to *p* map. We have provided initial validation of this technique against manual contours performed by experienced clinicians.

**Advances in knowledge:**

This novel automated technique to generate *p* maps has the potential to individualise radiation treatment volumes and act as a decision support tool for the treating oncologist.

## Introduction

 Glioblastoma multiforme (GBM) is an aggressive form of primary adult brain tumour, responsible for more average years of life lost than any other common adult malignancy.^1^ Survival for the population of patients diagnosed with GBM in England remains bleak, with over 70% of patients dying within 12 months of diagnosis.^2^ However, fit patients who undergo aggressive multimodal therapy (total macroscopic surgical resection, followed by chemoradiation) therapy may live for longer periods of time, with 2-year survival rates of 24% being reported in meta-analysis.^3^ This is relevant as a proportion of these patients are now living long enough to express the risk of radionecrosis and neurocognitive dysfunction related to chemoradiation therapy.

Glioblastoma is characterised by macroscopic and microscopic infiltration into adjacent brain tissue, by contiguous tumour spread, white matter migration, or spread through the subventricular zone. For this reason, generous clinical target volume (CTV) margins are added to the edge of the gross tumour volume (GTV), generally taken as the post-operative resection cavity, at the time of radiotherapy. Current European society for radiotherapy and oncology (ESTRO) consensus guidelines borne out of analysis of patterns of tumour recurrence following chemo-radiation therapy suggest that a 2 cm isotropic CTV margin is adequate to cover over 90% of tumour recurrences.^4,5^ Radiation therapy oncology group (RTOG) guidelines converge on a similar 2.5–3 cm margin.^6^

Diffusion tensor imaging (DTI) is a technique for imaging of white matter connectivity in the brain. We have previously demonstrated that it can be utilised to image subclinical tumour spread with high degrees of sensitivity and specificity, and patients can be stratified into prognostic phenotypes based on the extent of tumour infiltration as assessed by DTI.^7,8^ The DTI abnormality can predict the sites of tumour progression^9^ and decomposition of the diffusion tensor into isotropic(p) and anisotropic(q) components can provide spatial maps of tumour infiltration zones which correlate to the time of tumour progression.^8^ The p map is a imaging correlate for the infiltrative edge of the tumour, and therefore a surrogate for the CTV.^10^ For radiotherapy treatment planning, utilisation of DTI to individualise treatment permits a potential 40% reduction of CTV. Other studies have demonstrated the safety and efficacy of non-individualised reductions in CTV.^11^ Thus utilisation of DTI data at the time of radiotherapy planning might serve two distinct functions. The first is to reduce the irradiated volume of brain in patients with locally invasive tumours, with consequent reduction in the risk of cerebral radionecrosis and potential reduction in long term neurocognitive deficit. The second is to stratify patients according to risk of tumour infiltration and expected survival. At a practical level, integration of DTI data into conventional treatment planning systems is non-trivial and resource intensive due to the high dimensionality of the image data sets.^12^

In this paper, we demonstrate that DTI decomposition maps by principal component analysis can be combined with automated image segmentation to generated peritumoral tumour infiltration zones. Comparison of these volumes with manually segmented p maps, considered as a surrogate of conventional isotropic CTVs, demonstrates its potential to individualise radiation treatment volumes and act as a decision support tool for the treating oncologist.

## Methodology

**Figure 1.**
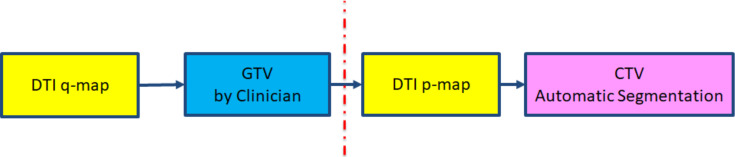
The framework of the proposed model in measuring CTV using DTI p and q maps. CTV,clinical target volume; DTI, diffusion tensor imaging; GTV, gross tumour volume.

The framework of the proposed approach is shown in Figure 1. This allows for easier contouring of the p, as a surrogate of CTV, for treatment planning based upon manually segmented q maps. The steps for this are:

1. The GTV is manually segmented from DTI q map, this will be the initialisation contour for level set.
2. Segmented GTV is superimposed on DTI p map.
3. Level set automatic segmentation modifies the superimposed GTV to include information from p map, this will incorporate the infiltrative edges.

In this paper, Chan-Vese level set segmentation model is applied. The reason of choosing this model is because of the robustness of this model on either conventional MRI or only DTI.

### Patient cohort, imaging and image processing

We utilised image data from a preoperative multimodal imaging study, providing data from 50 patients (mean age 58.2, range 31.4–71.6 years; 33 males, 17 female) with a histologically confirmed diagnosis of Glioblastoma. Written informed consent for research use of imaging was obtained for all patients, and was approved by the local research ethics committee (10/H0308/23). All patients were imaged at 3 T using a Magnetom Trio scanner. (Siemens Healthcare, Erlangen, Germany), using a standard 8-channel receive head coil and transmission on the body coil. DTI data were acquired using a single-shot echo-planar sequence (TR/TE 8300/98 ms; flip angle 90°; FOV 192 × 192 mm; 63 slices; no slice gap; and voxel size 2 × 2×2 mm) with multiple *b*-values (0, 350, 650, 1000, 1300, and 1600 s=mm^2^) scanned in 13 directions.

DTI images is an imaging technique aiming to the mobility of water within the brain. Furthermore, derived parameters from the tensor reconstruction have shown to be more sensitive than *T*_2_ weighted imaging in differentiating the gross tumour mass from the tumour in ltration.^8^ These derived quantities are the isotropic and anisotropic components of the diffusion tensor, respectively called p and q, and de ned respectively as follows. As the tensor flow is written as a 3 × 3 matrix for each voxel of the image, with eigenvalues $\lambda_{1}$ , $\lambda_{2}$ and $\lambda_{3}$ , then:

(1) $$\left\{ \begin{array}{l} isotropic\;component:p=\sqrt{3D}\\ anisotropiccomponent:q=\sqrt{{\left(\lambda_{1}-D\right)}^{2}+{\left(\lambda_{2}-D\right)}^{2}+{\left(\lambda_{3}-D\right)}^{2}} \end{array}\right.$$

where *D* is the mean diffusivity $D=\frac{1}{3}(\lambda_{1}+\lambda_{2}+\lambda_{3})$.

**Figure 2.**
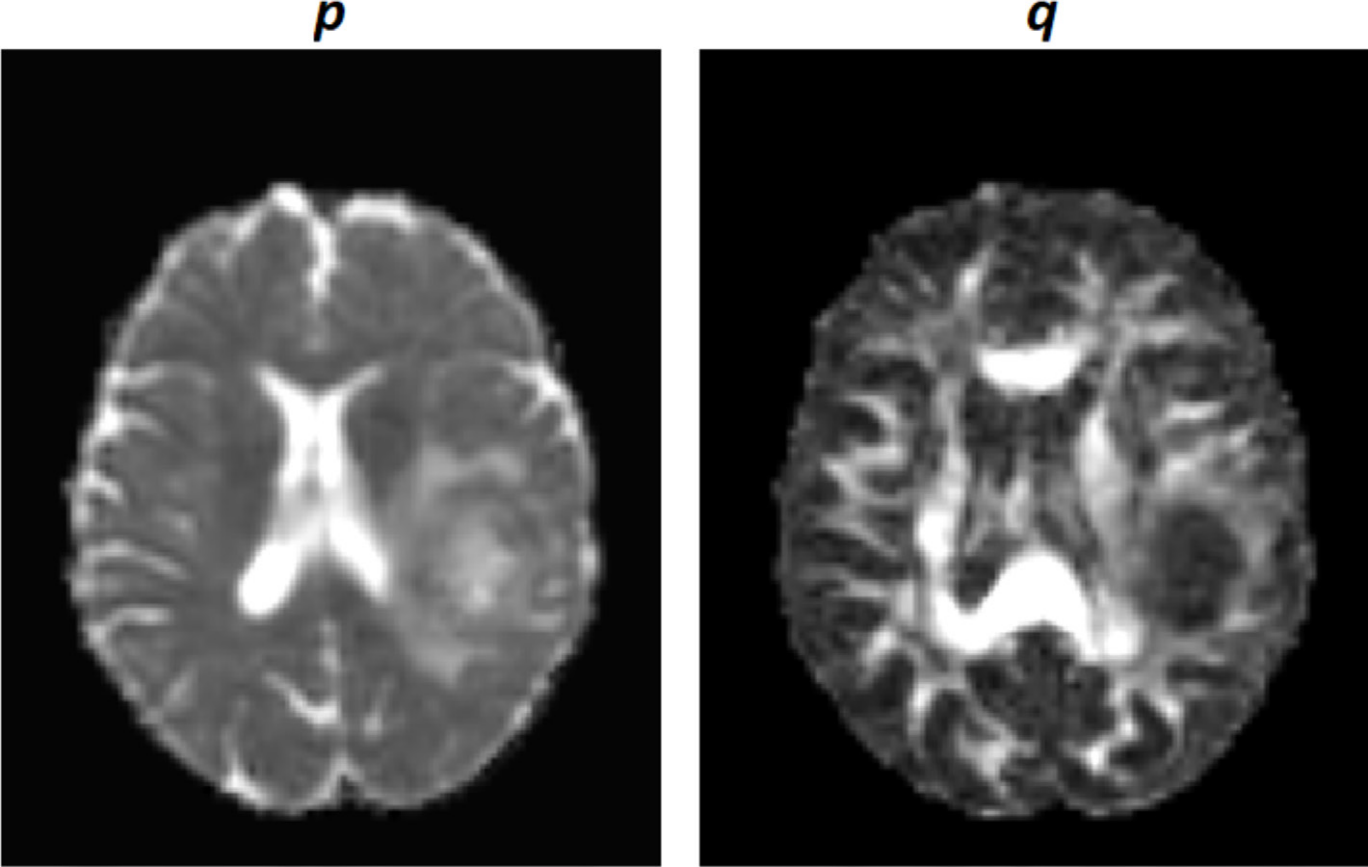
DTI-p isotropic component (left) and matching DTI-q anisotropic component (right). DTI,diffusion tensor imaging.

Figure 2 visualises the difference of p and q maps, where it shows the p-map is a surrogate for the extent of infiltrative tumour.

### Segmentation of regions of interest for analysis

Gold-standard manual segmentation of the preoperative GTV and CTV, together with p and q maps were performed by expert clinicians for all patients in the data set using the three-dimensional (3D) slicer application.^13^ The manual ROIs were independently contoured by the three observers: a neurosurgeon with >8 years of experience (CL), a neurosurgeon with >9 years of experience (JLY), a researcher with >4 years of brain tumour image analysis experience (NRB). The manual segmentation was blinded to the model construction and validation phases. The inter-rater reliability testing was performed using similarity coefficient scores to assure consistency among the observers.^12,14^

### Level set segmentation

Level set methods was developed in 1988 by Osher-Sethian^15^ to improve the shortcomings of the Snake technique introduced by Kass in 1987.^16^ Level set methods are a form of active contour which has the freedom of movement inside an image until it converges to the desired boundary/region. The level set needs to be initialized as a boundary. It was initially introduced as a Eulerian formulation of a propagating front, which moves with the speed F perpendicular to the curve. Level set’s front Γ(t) at any time t and each point (x,y) *,* propagates implicitly as the zero-crossings of a level set function (x,y,t) by the relationship:

(2) Γ(t)={(x,y)|ϕ(x,y,t=0)=0}

**Figure 3.**
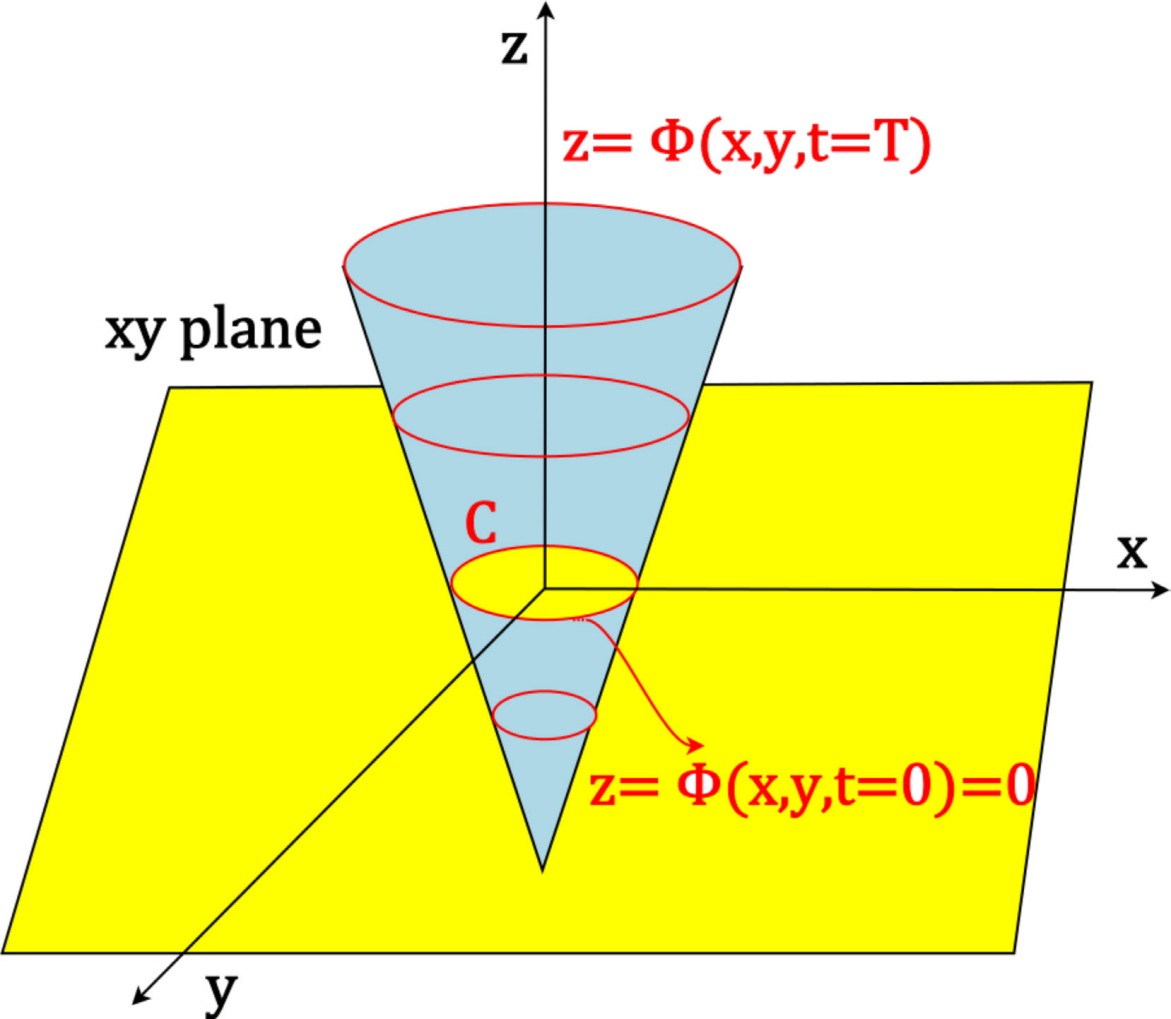
Level set function in blue, and zero level set surface in yellow.^17^

Figure 3 visualises the level set formation.

It adjusts the height of the z function which vanishes the topological problem. Such a level set is a growing or shrinking contour based on curvature-dependent speed for propagating fronts. It uses Hamilton-Jacobi equations to reconstruct complex shapes.

(3) z=ϕ(x,y,t)

ϕ(x,t=0)is the zero-level set contour which should be initialised as a contour to be the singed distance function.

### Two-phase Chan-Vese level set segmentation model

Chan-Vese proposed based on Mumford-Shah functional^18^ as two-phase level set method without edges.^19^ The main concept of this model was the improvement of energy minimization problem which is based on the mean intensity values in each region of the level set (inside or outside in two-phase), $c_{1}$ and $c_{2}$, defined as:

(4) $$c_{1}=\frac{\int_{\Omega }^{}\left(1-H\left(\phi \left(x,y\right)\right)\right)\left(I\left(x,y\right)\right)d_{x}d_{y}}{\int_{\Omega }^{}1-H\left(\phi \left(x,y\right)\right)d_{x}d_{y}}$$

(5) $$c_{2}=\frac{\int_{\Omega }^{}(H(\phi (x,y)))(I(x,y))d_{x}d_{y}}{\int_{\Omega }^{}H(\phi (x,y)){d_{x}d}_{y}}$$

*H* is the Heaviside function,

(6) $$H\left(x\right)=\left\{ \begin{array}{l} 1ifx\geq 0\\ 0otherwise \end{array}\right.$$

At each iteration, the values of $c_{1}$ and $c_{2}$ , change and must be recalculated based on the level set of a new region to calculate a new speed function as:

(7) $$\begin{array}{rl} F\left(c_{1},c_{2},\phi \right)= & \int_{\Omega }\left({\left(u_{0}-c_{1}\right)}^{2}H\left(\phi \right)\right)d_{x}d_{y}+\\ & \int_{\Omega }({\left(u_{0}-c_{2}\right)}^{2}\left(1_{H}\left(\phi \right)\right)d_{x}d_{y}+\\ & \int_{\Omega }|\nabla H\left(\Omega \right)|) \end{array}$$

The Chan-Vese level set evolution equation is as follow where represents a one-dimensional Dirac function.

(8) $$\frac{\partial \phi }{\partial t}=\delta \left(\phi \right)\left[\nu div\left(\frac{\nabla \phi }{|\nabla \phi |}\right)-{\left(u_{0}-c_{1}\right)}^{2}+{\left(u_{0}-c_{2}\right)}^{2}\right]$$

## Results

**Figure 4.**
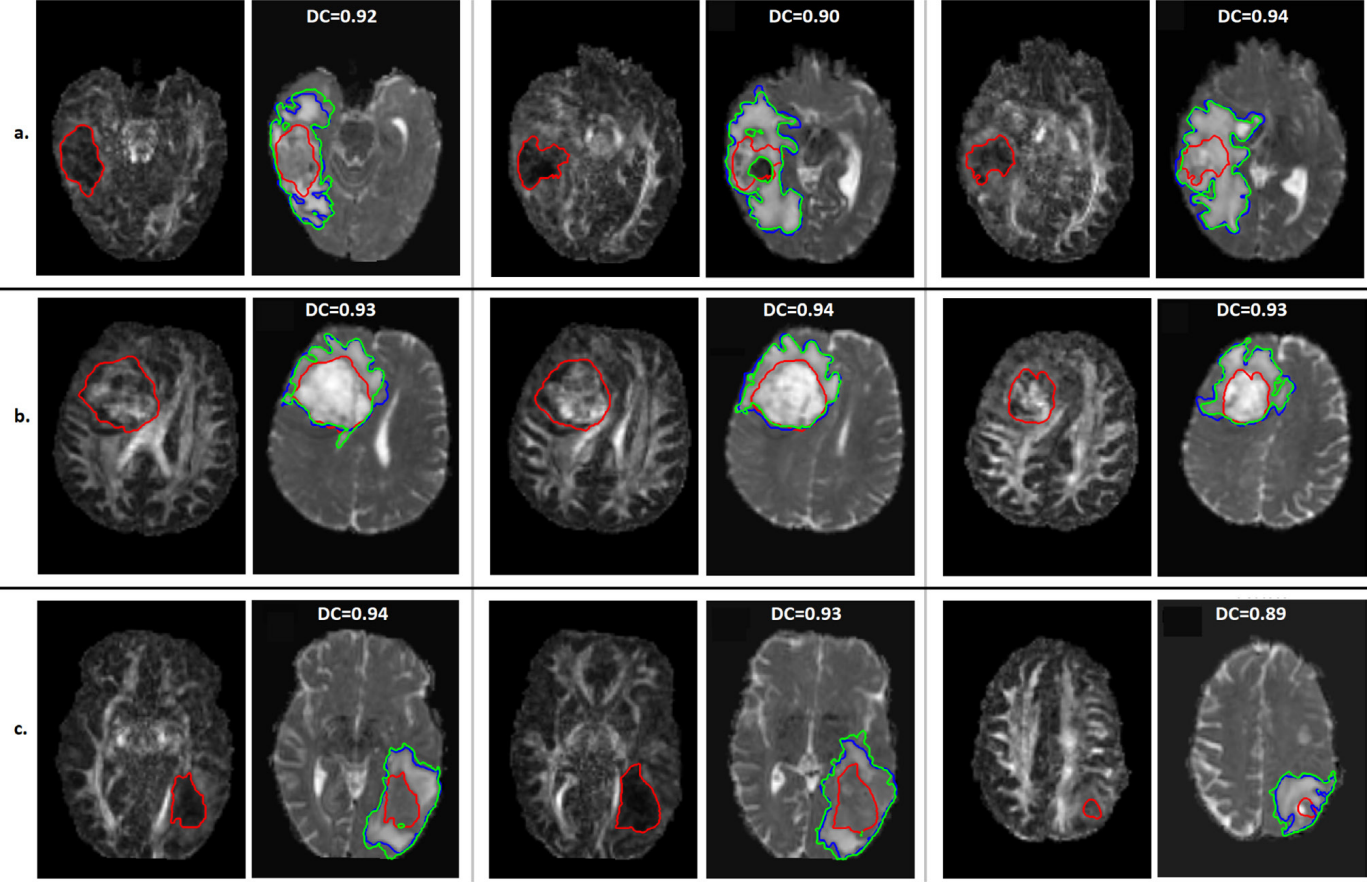
Level set segmentation result on q-map to estimate CTV during radiotherapy treatment for different slices of 3 patients, (a) Patient 1, (b) Patient 9 and (c) Patient 10. The relevant q-map for each slice is showing the difference and improvement in understanding tumour from GTV on q-map (initial contour of level set) to CTV on p-map (our segmentation). Red contour is GTV/ground truth on q-map which is used to initialise level set on p-map. Blue contour is the manual segmentation on p-map which is used as the gold standard for CTV boundary and to compare the result of our segmentation. Green refers is the outcome of our segmentation on q-map to measure CTV. CTV,clinical target volume; GTV, gross tumour volume.

p and q images have the volume of 240 × 330 × 23 pixels and a pixel size of 0.977 x 0.977 mm in nifti format. The manual segmentation/ground truth which refers to the GTV on q-map was used to initialise level set and tries to segment p maps which refer to CTV. Figure 4 visualises the information about the status of tumour to the clinicians for three slices for 3 example patients out of 50 cases. The expansion of tumour boundary from q-map to p-map is clearly visible and the Dice coefficient (DC) showed a similarity of greater than 90% on these slices to their relevant ground truth.

**Figure 5.**
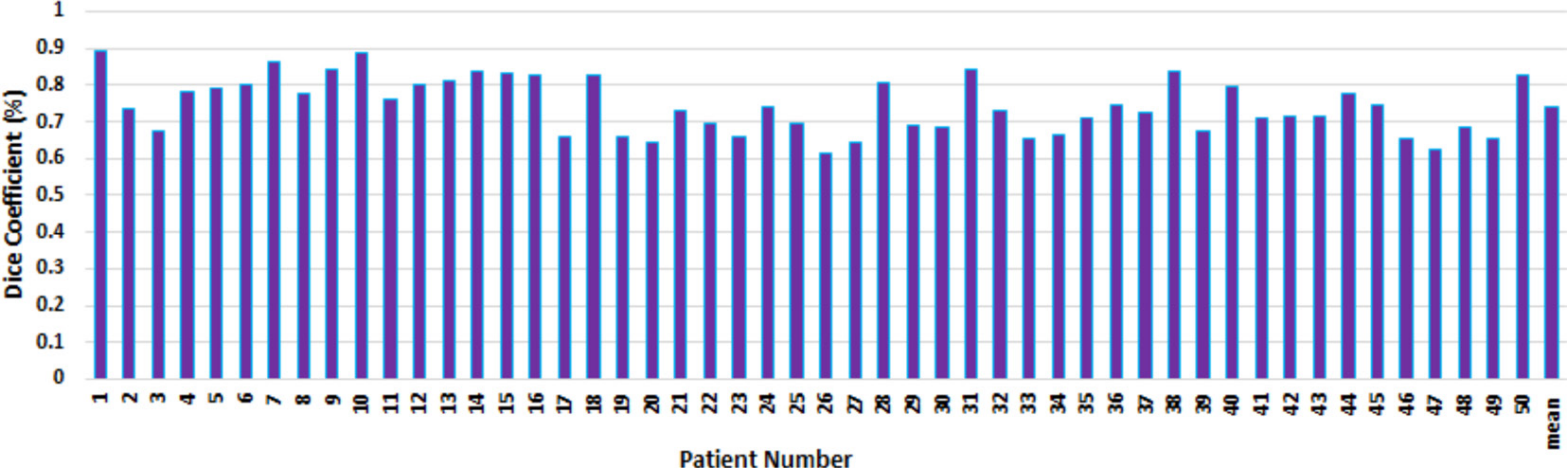
Dice coefficient comparison between the main CTV on p-map and Chan-Vese level set model. CTV,clinical target volume.

In order to evaluate the reliability of the proposed model, we seek to demonstrate that the expansion of tumour volume on p-mask can be used to measure CTV automatically. In Figure 4, blue contour is the manual segmentation on p-map which is used as the gold-standard for CTV boundary and to compare the result of our segmentation. The green contour refers to the outcome of our segmentation on q-map to measure CTV using Chan-Vese level set model. Figure 5 shows the DC between the manual segmentation on p-map and our segmentation for different patients on average of their slices.

Concerning conforming indices, the best agreement was determined with a mean score of 74% across all 50 patients’ average DC with the standard deviation of 7%. When the initial contour was done manually, further analysis revealed that the average CTV contouring time of proposed model was 0.5 s which is less than the overall average time in planning practice done by clinicians. The consistency in the proposed model’s segmentation among all different patients shown in this figure is a great indication to reduce the inter- and intraobserver variability in GTV and CTV contour generation.^20^

## Discussion

Segmenting GBM and its recurrence boundaries is a very challenging area as the image modalities are restricted to conventional MRI before treatment. Although the outcome of treatment in GBM demonstrates the lack of information on used MR images, the proposed approach in analysing GBM tumours based on DTI p and q-maps images would help the clinicians to better estimate the location and size of the tumour as well as recurrence area of tumour which is not really visible to the conventional MR images. The standard treatment for GBM patients is still surgery. Surgery is also of use, because it provides tissue specimens to evaluate, so that an appropriate treatment can be defined based on the accurate evaluation of the tumour, biologically. A better individualisation of treatment approaches requires a more precise understanding of the recurrence regions of GBM. However, there are some real limitations to surgery. The proposed model would give an indication to the clinicians about the potential areas of tumour using radiotherapy. The great possibility in generating accurate CTV using DTI and specificity using p and q maps which are completely different, would lead into better merging the isotropic and anisotropic information of the brain white tracts. The proposed CTV modelling improves using the p and q maps segmentation.

Our algorithm can reliably generate DTI sequence derived p maps from manually segmented q maps. Automatic segmentation of p and q abnormalities can be performed robustly. As we have previously demonstrated that the p map represents the infiltrative margin of the tumour, it acts as a biological surrogate for the CTV concept of microscopic disease extent used in radiotherapy planning. The volumes derived from our automated tool are highly comparable to the manual CTV contours derived by experts using a geometric expansion round the tumour of 2 cm in the case of the ESTRO ACROP (Advisory Committee on Radiation Oncology Practice) guidelines or the 2.5 cm in the case of the RTOG guidelines.^21^ As suggested by Price et al,^22^ isotropic p component appears to increase with tumour infiltration due to the increased tissue water from vasogenic oedema. This is a very common finding in GBM imaging and automation of part of the analysis for this oedematous, infiltrated region may have further research and clinical applications.

Due to limitations on scanning time, DTI acquisitions are noisy when compared to clinical T1 and T2 sequences. The Chan-Vese model performs robustly in the presence of this noise due to its ability to utilise global properties such as regional image intensity. Also, the level set algorithm considers the interrelation of p and q (defined by the equation), since it initializes from the q region to derive an accurate segmentation of p. The robust results of the algorithm, comparable to manual segmentation by experienced clinicians, suggests that this iterative level set method may perform well in the GBM population with further parameter tuning.

Whilst manual segmentation of image maps by expert observers is considered the gold-standard, it is well appreciated that manual segmentations are subject to significant inter- and intraobserver variability. Current clinical management of GBM is very firmly based on the convention of using *T*_1_ weighted sequences with contrast and FLAIR sequences. Use of these without reference to the information available from DTI-derived sequences can considerably underestimate the true tumour volume. By automating the conversion of a q region to a p region, this additional information may also assist in guiding the treatment plans of clinicians considering resective surgery or radiotherapy, *e.g.* to establish whether or not total macroscopic excision of the tumour is feasible.

In radiotherapy, the GTV contours are manually delineated way for each patient. In this work, we have not used user-input to find the location of the GTV. Using the proposed technique would provide personalised CTV solutions for each patient at the same time. This would influence the resulting radiation dose calculation and final treatment toward the desired direction of harming less healthy cells and radiating more cancerous cells. Also, this method would significantly reduce the CTV contouring task complication time. Thus, the use of such automatic contouring tools can be encouraged in radiotherapy planning software.

We know that the central concept of the CTV is to encompass subclinical tumour infiltration. Given that our previous work demonstrates the validity of the p-map as a surrogate for tumour infiltration, we consider that the automatically generated CTV has value as the basis of personalising the CTV. Clearly, personalisation of the CTV that differs significantly from conventional margins would require validation in a prospective clinical study but existing data on patterns of treatment failure would support such an approach. Furthermore, there may be other anatomical zones at risk of tumour infiltration that are not adequately visualised using DTI (*e.g.*, the adjacent subventricular zone) and careful consideration would need to be given to the handling of these regions in an individualised margin protocol.

Personalised-based CTV contours were significantly more heterogeneous than the automatic generated ones in radiotherapy practice. DC resulted in more consistent irradiated volumes between p region ground truth and Chan-Vese level set model. The authors speculated the consistency of personalised-based CTV contours shows the consistency in tumour sites, potentially resulting in geographic miss in dose delivery, which could hamper local control for individual patients.

The results in the proposed method is influenced by a number of variables, such as the parameter settings as the type of image acquisition and reconstruction methods. While still standardising our image data and normalising them can ease this matter and help towards more generalization of the contours. However, the algorithm could be modified to use input information regarding the location of GTV to further improve the speed and accuracy. Also, furthermore the method can be extended into 3D modelling to automatically generate volumes based on level set growth in 3D space.

## Conclusions

We presented a method for automatic segmentation of the tumour infiltration boundary using DTI and tensor decomposition. The first application is in decision support for assessment of MR images for surgery planning and therapy response, such as for data analysis with the PRaM-GBM multicentre clinical trial (ethics approval number: 16/EE/0476). The second is for radiotherapy target volume definition. The technique offers the opportunity to generate an automated yet individualised CTV for treatment planning, rather than the current CTV which is a simple geometric expansion that is driven by an assessment of tumour infiltration across a population of patients. Such a CTV might be used as the basis of an individualised dose intensity-modulated radiation therapy dose painting strategy. Further cross-validation of the segmentation technique is required to confirm its utility.
